# Technical assessment of the NDI Polaris Vega optical tracking system

**DOI:** 10.1186/s13014-021-01804-7

**Published:** 2021-05-12

**Authors:** Giovanni Fattori, Antony John Lomax, Damien Charles Weber, Sairos Safai

**Affiliations:** 1 Center for Proton Therapy, Paul Scherrer Institute, Forschungsstrasse 111, 5232 Villigen, Switzerland; 2 Department of Physics, ETH Zurich, 8092 Zurich, Switzerland; 3 Department of Radiation Oncology, University Hospital Zurich, 8091 Zurich, Switzerland; 4 Department of Radiation Oncology, University Hospital Bern, 3000 Bern, Switzerland

**Keywords:** Optical tracking, IGRT, Breathing motion, Patient positioning

## Abstract

The Polaris product line from Northern Digital Inc. is well known for accurate optical tracking measurements in research and medical environments. The Spectra position sensor, to date often found in image guided radiotherapy suites, has however reached its end-of-life, being replaced by the new Vega model. The performance in static and dynamic measurements of this new device has been assessed in controlled laboratory conditions, against the strict requirements for system integration in radiation therapy. The system accuracy has improved with respect to the Spectra in both static (0.045 mm RMSE) and dynamic (0.09 mm IQR, < 20 cm/s) tracking and brings marginal improvement in the measurement latency (14.2 ± 1.8 ms). The system performance was further confirmed under clinical settings with the report of early results from periodic QA tests within specifications. Based on our tests, the Polaris Vega meets the quality standards of radiotherapy applications and can be safely used for monitoring respiratory breathing motion or verifying patient positioning.

## Introduction

Position tracking systems are an essential component of computer assisted interventions requiring intraoperative navigation of tools and therapeutic devices relative to the patient. Although largely adopted in surgical settings, with the widespread introduction of image-guided procedures in modern clinical workflows, their use has extended into other medical fields that profit from accurate measurements for patient registration. In a recent publication, we have reported on the use of such tracking systems to monitor breathing motion during radiation therapy [1]. Optical tracking has been favoured against electromagnetic solutions after two de-facto standard devices in the OEM market, i.e. Aurora and Polaris Spectra tracking systems from Northern Digital Inc. (NDI), have been compared under clinical settings [2].

The Polaris Spectra however has recently been pulled from the market after reaching product end-of-life and its successor, commercialised under the name Polaris Vega, has not yet undergone a thorough testing by the research community. With this contribution, we expand on our previous publication [2], assessing the performance of Polaris Vega under the same testing protocol, which covers the key measurements for the clinical integration of optical tracking technologies. The tests have a general validity, even though the study design follows the guidelines and principles of AAPM TG 147 report for quality assurance (QA) of nonradiographic localisation systems in radiotherapy [3]. Results are similarly discussed based on our early experience of use for breathing motion monitoring in clinical settings.

## Connections and programming interface

The test unit under consideration was a standard Polaris Vega device without the radiation hardness option, wired to the Ethernet interface of a computer running Scientific Linux 6. The application programming interface (API), released with previous Spectra models (IL-1070101 R5), was used to communicate with the new hardware by creating a virtual serial connection attached to the TCP port of the sensor on the network interface. All measurements were taken tracking individual (*stray*) passive markers at 60 Hz and using high-performance binary data transmission format.

## Spatial measurement reproducibility and thermal drift

**Fig. 1:**
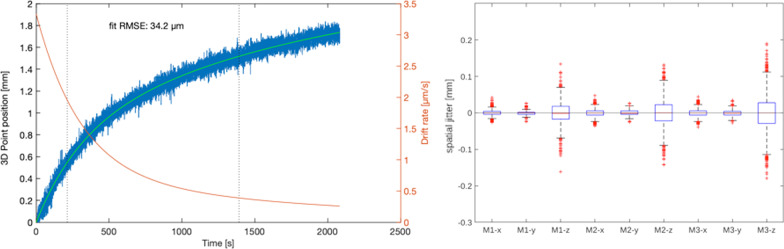
On the left panel, the 3D position measurement thermal drift over 2000s after camera cold start. Raw data in blue are fit with a two-terms exponential decay plotted in green and its first derivative drift rate is in orange. The two vertical dotted lines indicate the half times of the fast and slow drift components. The measurement jitter on individual direction components (x,y,z) is displayed on the right panel, for three points located at approximately 150 cm (M1), 151.5 cm (M2) and 175 cm (M3) from the camera sensor

Measurement reproducibility on stationary optical markers, also known as spatial jitter, has been measured after the transitory thermal drift, which was observed to follow a two-term exponential decay of the form:

$$Drift\left( t \right) = Span_{Fast} *\left( {1 - e^{{ - {\raise0.7ex\hbox{$1$} \!\mathord{\left/ {\vphantom {1 {\tau_{Fast} }}}\right.\kern-\nulldelimiterspace} \!\lower0.7ex\hbox{${\tau_{Fast} }$}} * t}} } \right) + Span_{Slow} *\left( {1 - e^{{ - {\raise0.7ex\hbox{$1$} \!\mathord{\left/ {\vphantom {1 {\tau_{Slow} }}}\right.\kern-\nulldelimiterspace} \!\lower0.7ex\hbox{${\tau_{Slow} }$}}* t}} } \right)$$

where $Span_{Fast} = 0.804\,{\text{mm}}$, $Span_{Slow} = 1.445\,{\text{mm}}$, $\tau_{Fast} = 307.34\,{\text{s}}$ and $\tau_{Slow} = 2007.5\,{\text{s}}$, and the half-time drifts of the testing device were equal to 213.03 s and 1391.5 s for fast and slow components respectively (Fig. 1—left panel).

With the system in stable condition, after more than 2500 s of warm-up time, the position of three markers located at distances of ca. 150 cm (M1), 151.5 cm (M2) and 175 cm (M3) from the camera were measured for 1 min, replicating the setup introduced in our previous publication. The root mean square (RMS) error of 3D measurements increased from the 0.029 mm of M1 to 0.045 mm of M3, due to the larger variance on the longitudinal dimension (z) as a function of the camera-point distance (Fig. 1—right panel).

Additionally, the full data recording acquired to assess thermal drift has been used to evaluate the reliability of the camera sampling-rate over long acquisitions, which was found to match accurately the nominal values (60 Hz) with 16.67 ± 0.6 ms time interval between subsequent data frames.

## Dynamic tracking error

**Fig. 2:**
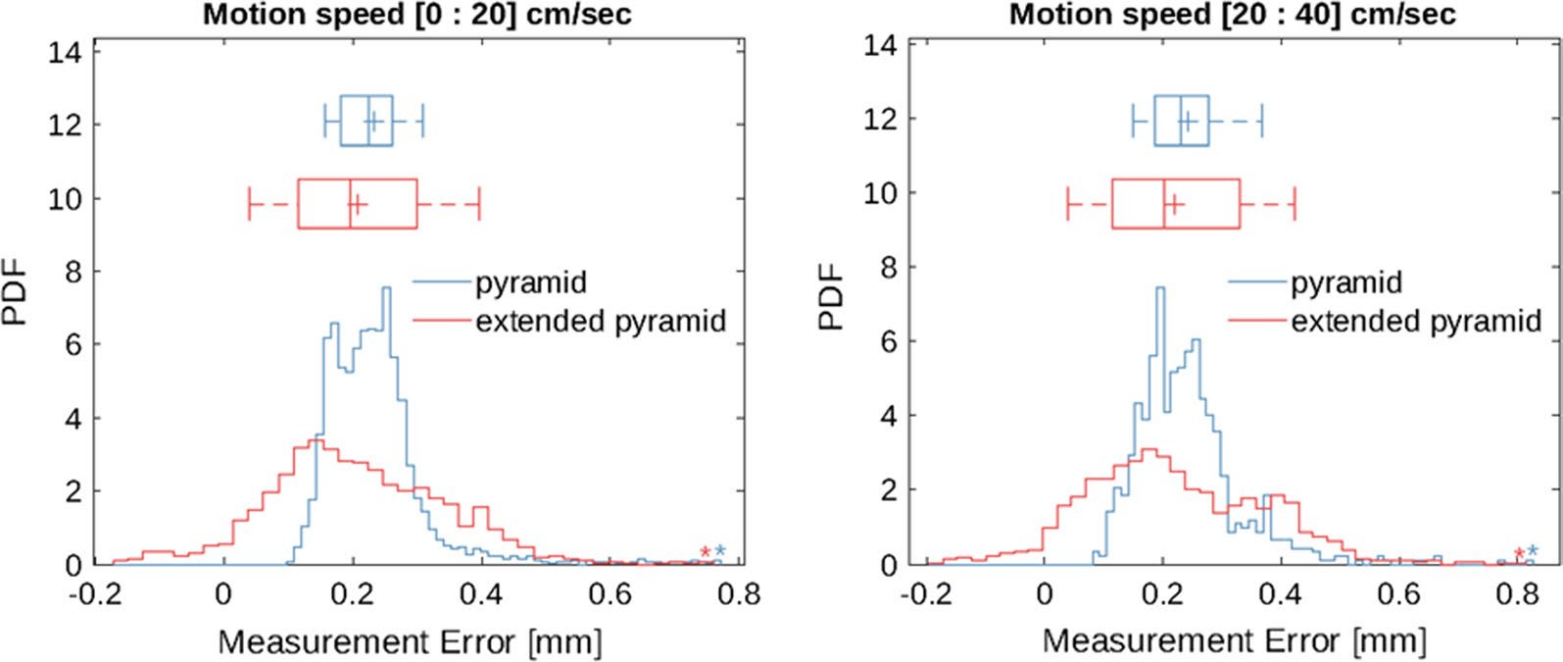
Probability density function of dynamic tracking error for Pyramid and Extended Pyramid measurement volumes for two speed classes, below 20 cm/s (left panel) and between 20 and 40 cm/s (right panel)

The dynamic tracking error has been assessed for the two measurement volumes that the system supports, *Pyramid* covering up to 2.4 m from the sensor and the *Extended Pyramid*, which reaches 3 m distances. The position of optical markers mounted on the NDI Rigid Body tool (Part number 8700339) were tracked during free hand motion and the variation in the distance between two points used as figure of merit for the dynamic measurement error. Results are reported for two classes of speed (Fig. 2). Below 20 cm/s the inter-quartile ranges (IQR) were 0.08 mm and 0.18 mm for the *Pyramid* and *Extended Pyramid* respectively and increased only marginally as a function of speed, with an IQR of 0.09 mm and 0.22 mm for the two volumes when tracking motion between 20 and 40 cm/s. All measurements were consistently off the 55 mm reference markers distance by 0.2 mm (median value), a bias attributable to the manufacturing tolerances of the phantom and sphere markers.

## Measurement latency

The Polaris Vega has also been benchmarked for measurement latency against an analogue distance sensor (FADK 14U44790/IO, Baumer Electric AG, Frauenfeld, CH) which tracked the sinusoidal motion of the Anzai respiratory phantom (AZ-733V, Anzai Medical Co., Tokyo, J). Data from the optical tracking system and the digitised sensor signal (PCI-6221, National Instruments Corp., Austin, US) were recorded and compared using common timestamps. The phase difference of the Fourier transform at the fundamental frequency of the phantom motion (0.25 Hz) was 14.2 ± 1.8 ms over three repeated measurements lasting 60 s each.

## QA of dynamic localisation accuracy in clinical settings

Spatial and temporal localisation accuracy of optical tracking systems are regularly tested as part of our QA program for respiratory gating equipment [1]. Since the clinical integration in our proton therapy department, the device has been verified on a monthly basis, tracking the sinusoidal motion of a programmable breathing phantom (QUASAR™ 100-1010, Modus Medical Devices, London, CA). Measured motion range and period acquired during 10 min of acquisition are compared with the nominal displacement of the chest wall platform by 1 cm amplitude in 5 s. The measurement follows a geometric calibration that maps the camera coordinate system into world coordinates, hence allowing to track the vertical component of the phantom motion. Maximal absolute discrepancy in measuring breathing amplitude and period after 5 months of operation are respectively equal to 0.32 mm and 18 ms.

## Discussion

With Polaris Spectra now taken off the market, Vega is the new reference technology for optical measurements from Northern Digital Inc. The new model improves upon its predecessor with lower jitter, reduced distortions in dynamic measurements and the inclusion of a network interface. Otherwise, the second-generation optical tracking systems are similar to the Spectra sensors, with a comparable footprint and no major design updates that, together with the backwards compatible API, allows for almost seamless integration into existing environments which already use NDI technology.

As for jitter measurements, a larger uncertainty in the longitudinal direction is inherent to the optical triangulation procedure, and Vega is no exception following the same trend observed for Spectra types [2]. The root mean square is however reduced from 0.06 mm of the previous model to 0.045 mm, as measured at our laboratory with the test unit. It should be noted however that the substantially large measurement drift observed with our testing device is device specific, and should not be taken as a reference value of the product line. More relevant is the lower measurement error in dynamic tracking, which halves the older model’s uncertainty for objects moving below 20 cm/s. With object speeds up to 40 cm/s, a value that is still relevant for medical radiotherapy applications, no impact has been found on measurement quality. Finally, the latency reduction of about 10% is worth noting, albeit if only of interest for applications with strict timing requirements. We reported on the measurement of individual passive markers rather than the more demanding requirement of tracking the position of rigid tools which may indeed affect the system speed. Nevertheless, our commissioning data is below the 18 ms latency which is conservatively specified by the manufacturer [4] and well within 100 ms limit recommendation given in the AAPM TG142 report for LINAC applications [5].

Our clinical QA setup replicates the IGRT use case, where arbitrarily positioned cameras are calibrated with respect to the treatment isocentre. The geometric calibration error adds to the limited reproducibility of the phantom vertical motion, which in our model version is obtained as a result of a mechanical rolling bearing on a rotary cam. Under this condition, the dynamic localisation in clinical settings has proved to be affected by larger errors compared to the commissioning test run in laboratory conditions. Nevertheless, the reported spatial localisation error is well below the 2 mm recommendation as per TG 147 and similarly the temporal accuracy is within specifications [3].

As concerns the technical performance for breathing motion monitoring, the system has improved with respect to the Spectra for measurement accuracy in static and dynamic conditions, while bringing marginal improvement for measurement latency. As such, we conclude that the system can be used safely in radiotherapy applications for monitoring respiratory breathing motion, and its use to verify patient setup can benefit from its improved accuracy in static measurements. Being a point-based tracking system, it is hard to make a direct comparison with more sophisticated surface guided radiotherapy solutions [6], which are specifically designed to reconstruct patient body surface as a cloud of dense points. On the other hand, the streamlined stereo-photogrammetric approach to marker localisation provides high accuracy measurements at low latency for demanding applications where timely reaction is the key for synchronising radiation therapy with patient motion or intraoperative navigation.

## Data Availability

The datasets used and/or analysed during the current study are available from the corresponding author on reasonable request.

## Abbreviations

API: Application programming interface
IGRT: Image guided radio therapy
IQR: Interquartile range
LINAC: Linear accelerator
OEM: Original equipment manufacturer
OTS: Optical tracking system
RMSE: Root mean square error
TCP: Transmission control protocol
TG: Task group
